# Printable and flexible photodetectors via scalable fabrication for reading applications

**DOI:** 10.1038/s44172-022-00041-4

**Published:** 2022-12-01

**Authors:** Georgios Bairaktaris, Fasihullah Khan, K. D. J. Imalka Jayawardena, David M. Frohlich, Radu A. Sporea

**Affiliations:** 1 Advanced Technology Institute, University of Surrey, Guildford, GU2 7XH UK; 2 Centre for Integrative Semiconductor Materials, Swansea University, Swansea, SA1 8EN UK; 3 Digital World Research Centre, University of Surrey, Guildford, GU2 7XH UK

**Keywords:** Electrical and electronic engineering, Techniques and instrumentation

## Abstract

Printing techniques have been widely adopted in the fabrication of flexible electronic components. However, its application is still limited in complex control and communication circuitry due to the low performance and low fabrication uniformity amongst printed devices, compared to conventional electronics. Thus, the electronic systems in real-world applications are hybrid integrations of printed and conventional electronics. Here we demonstrate a low-cost, low-complexity, fully-printable flexible photodetector that can withstand over 100 1 mm-radius bending cycles using a simple and scalable two-step fabrication process. The prototypes are implemented in an augmented book system to automatically detect the ambient light through optical apertures on paper of a printed book, and then transmit the information to an adjunct device. This technique demonstrates the utility of low-cost materials and processes for robust large area sensing applications and could act as a gateway to pertinent multimedia information.

## Abstract (web summary)

Bairaktaris and colleagues developed a printable flexible photodetector using a simple, scalable fabrication process. The photodetectors were demonstrated in practice in an augmented paper system. This low-cost technique could be also applied in robust and large-scale user interfaces.

## Introduction

Technology is continuously incorporated within our everyday life, and printed electronics play a crucial role for a successful and seamless future integration into compelling functional systems^1–6^. A challenging application is represented by intuitive user interfaces, which can bridge the gap between the analogue and the digital world, allowing users to control technology by interacting with electronically augmented physical objects^7^. The flexibility and conformability of these new types of electronics, as described above, allow for electronic systems to be incorporated in our everyday life without changing the way we interact with the objects around us^1,3,6–9^.

Printing techniques in combination with solution processing are widely adopted in research and development for the fabrication of electronic components such as transistors, diodes, and photodetectors^10^. The low-temperature process and the low-energy, additive nature of printing allows for more sustainable and environmentally friendly electronics, compared to conventional semiconductor processing techniques^2,10^. Additionally, these techniques are compatible with a wide range of flexible substrates (e.g., paper, polyethylene terephthalate, polyimide, polyethylene naphthalate), which allows devices to be made bendable and conformable to arbitrary shapes. Nevertheless, in many cases, this potential is diminished by the competing optimisation requirements of the functional materials, substrates, patterning techniques, and the throughput required to meet cost targets^1^.

Multiple systems in the literature utilise printing techniques for realising user interfaces^11–14^. Examples for applications involving printed electronics include PrintSense, a multimodal on- and near-surface sensing technique for planar, curved, and flexible surfaces, which is utilising a universal interdigitated electrode design, inkjet printed on a flexible substrate, for touch, proximity, pressure, and flexing sensing^15^. FlexTouch is a technique that allows long-range touch sensing interfaces beyond commercial touchscreens, using printed conductive traces as capacitive sensors for larger areas^16^. Multi-Touch Kit is a technique that is using printing on paper to enable multi-touch sensing on arbitrary shapes and surfaces^17^. These examples use the flexibility and low cost of printed conductors for rapid iterative prototyping of user interfaces that can be seamlessly embedded in physical objects.

One common feature amongst these examples is that the printed electronics used can only replace a finite number of conventional sensors and electronics, via relatively simple circuits. Complex control and communication circuitry cannot yet be replaced by printed equivalent circuits. This is mainly due to limitations such as the low performance (e.g., semiconductor mobility and transistor speeds), and low fabrication uniformity amongst printed devices, compared to conventional electronics, that challenge their adoption in commercially viable systems^1^. Adapting photolithography techniques for flexible substrates has allowed for ARM reduced instruction set computing (RISC) microcontrollers to be implemented on polyimide substrates^18^, which opens up many opportunities for future fully flexible real-life systems, while incurring a relatively large fabrication cost. When resorting exclusively to lower-cost printing techniques for functional circuits, even the latest attempts only realise limited functionalities and require additional, conventional electronic circuits to operate as intended in the application^19–22^. Therefore, at present, the hybrid integration of conventional and emerging electronics or techniques is the best solution for combining the advantages of printed components (e.g., flexibility, conformability, low cost, rapid prototyping) with the high performance and complex functionality of conventional electronics for realising practical systems for real-life applications^1,3^.

An elegant approach to moving the hybrid integration balance toward flexible and printed techniques is to utilise the specific advantages of emerging materials and processes, while making a large proportion of their limitations irrelevant for the application. In particular, holistic system design can reveal viable ways of utilising low-cost approaches despite intrinsic performance or variability limitations and has the potential greatly broaden the range of viable applications for these emerging technologies.

In this work, we report on application-specific printed photodetectors that are designed to detect ambient light through optical apertures on paper, in order to detect the open page of an augmented book^23^. The detectors are developed in a minimally complex way using two simple fabrication steps; demonstrate an on/off ratio of 20.9, sufficient to detect ambient light presence using a typical microcontroller’s analogue-to-digital converter (based on an ATmega328 MCU); and can withstand over 100 1 mm radius bending cycles without affecting the detection capability of the final demonstrator.

**Fig. 1:**
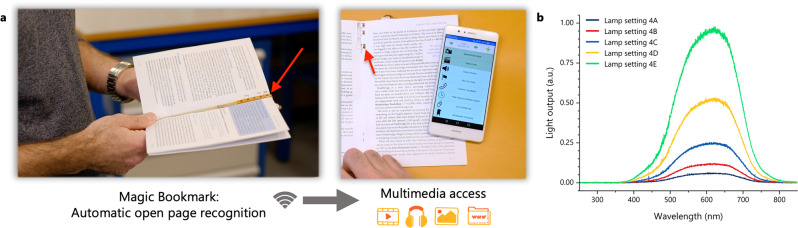
The augmented paper ecosystem and the test conditions. **a** Overview of the Magic Bookmark ecosystem; an electronically augmented bookmark is used on a printed book to automatically detect and transmit the open page to an adjunct device. **b** The emission spectrum of the microscope lamp that is used to assess the suitability of the developed photodetectors (settings refer to the lamp settings as outlined in Supplementary Table 1).

We have previously reported on our work on augmented paper which seamlessly links digital assets to printed content on paper, using a hybrid electronic device in the form factor of a book. In our latest development^23^, we created the Magic Bookmark concept (Fig. 1a), which utilises hybrid integration to create a flexible, electronically enhanced bookmark that automatically detects the open page of a book, and triggers relevant multimedia content on a nearby display. The hybrid Magic Bookmark is currently being used for the implementation of the Climate Domesday Book, a hybrid print-digital device that explores contemporary questions related to the climate emergency. We are now making advances towards a fully printable and flexible user interface by replacing the conventional photosensors of a hybrid system with the developed flexible and printable detectors, which have been designed specifically with the requirements of the second implementation of the Magic Bookmark demonstrator in mind. This implementation is using optical sensors to read an ambient light binary code through optical cut-outs on the pages^23^. A reference sensor is always exposed to allow for deviations in lighting conditions, and analogue-to-digital converters are used to read the output of each sensor because the photogenerated output generated under low lighting conditions might not be sufficient to trigger a microcontroller’s input threshold^23^. However, the developed detectors’ use can extend to any user interface that requires light or even touch inputs.

The fabrication simplicity and detection performance reported in this work allow for fully printable and flexible photodetectors made with mainstream functional materials to be easily incorporated within user interfaces. To our knowledge, this is the first demonstration withing the Human Computer Interaction (HCI) domain of systems that allow humans to interact with the digital world through physical objects, in which the sensing is achieved without complicated processing steps or relying on hybrid integration of conventional of-the-shelf sensors.

In the next section, we set the necessary requirements for light sensors used in user interfaces. Next, we outline the development or both oxide and organic semiconductor photodetectors and assess their suitability. Lastly, we demonstrate how these devices can be integrated and used in a real-life application as part of a functional hybrid system with fully-printable and flexible photodetectors for ambient light sensing.

## Results and discussion

### Application requirements

The key to utilising emerging technologies and devices in real-life is to understand the requirements of each class of applications and selectively address the technology challenges accordingly. The photodetectors developed in this work are optimised for user interfaces and in particular for the augmented paper ecosystem^23,24^. The premise for the detection is the necessary presence of incident light on the paper surfaces with which the user is interacting, and conversely, its absence from other parts of the augmented object, in this case, the other pages of the book^25^. In such applications the light source is either a type of indoor light (e.g., LEDs, halogen lamps) or outdoors sunny conditions. Therefore, the input spectrum we used to test the developed devices covers most of the visible wavelengths as seen in Fig. 1b. Additionally, the Magic Bookmark system relies on the detection of ambient light presence and uses a reference sensor to accommodate for deviations in operation between sensors within the array. This affords exceptional tolerance to manufacturing variability and ageing effects, greatly reducing the need for material, process, and performance optimisation. Instead, the detectors should simply be able to reliably distinguish between two states, dark (sensors covered by hand or paper) and light conditions (sensors exposed to light).

Two important properties for the photodetectors are manufacturability and sustainability. The fabrication process should be scalable, i.e., compatible with scale-up fabrication techniques such as roll-to-roll printing, eco-friendly using low-energy and minimal waste additive techniques, and the yield should be high to keep final production cost low. Finally, the photodetectors need to be flexible and conformable to various surfaces to allow for easy integration with everyday objects. For example, the Magic Bookmark application requires flexible sensors on the bookmark for the system to look and feel like a typical bookmark.

In the following sections, we outline the evolution and the main characteristics of the sensors. We then demonstrate our approach for integrating the developed devices with the existing augmented paper ecosystem.

### Oxide semiconductors – photodetectors structure

**Fig. 2:**
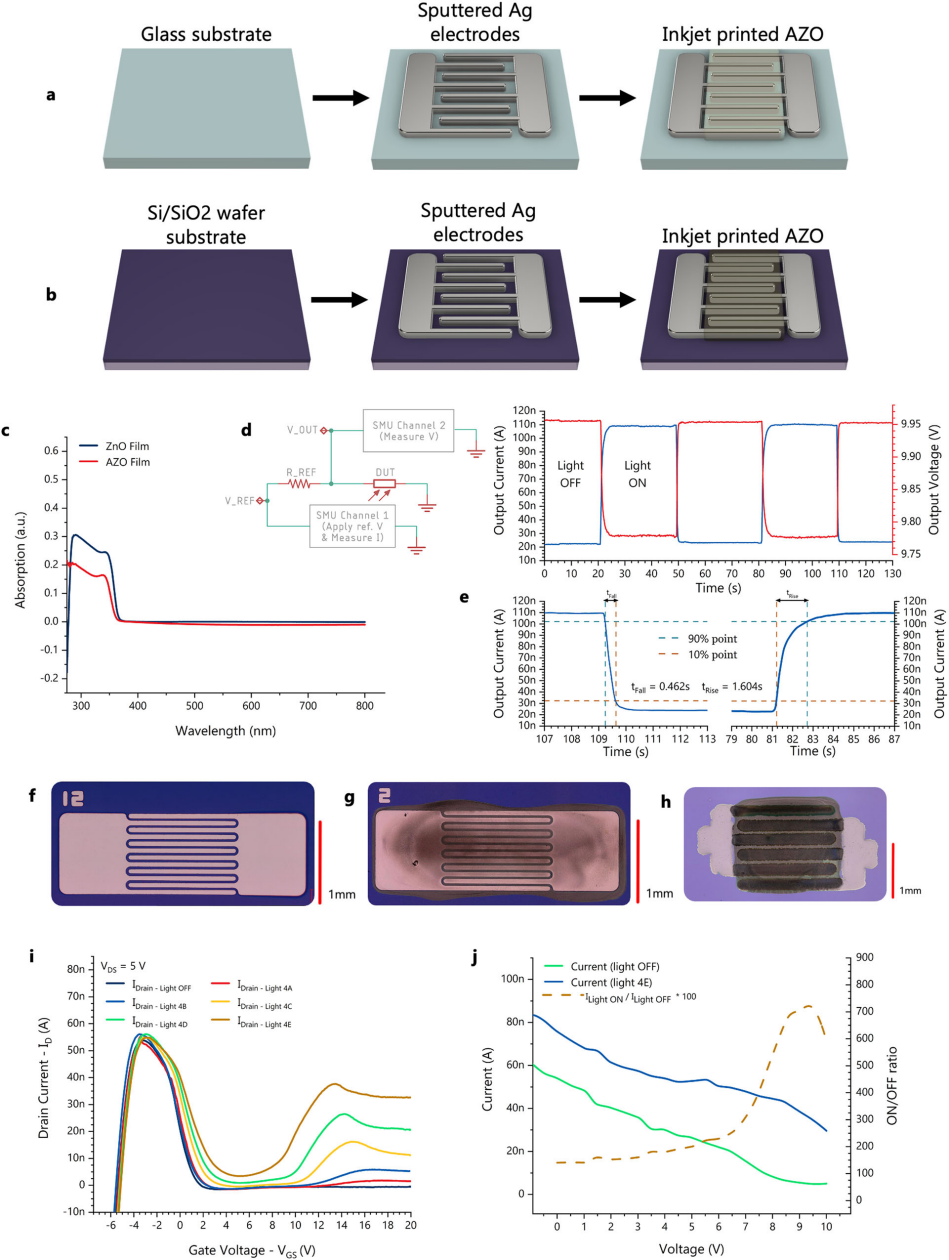
Electrical characterisation of the oxide semiconductor photodetectors. **a** Fabrication process for the Aluminum-doped Zinc Oxide (AZO) devices on glass substrates. **b** Fabrication process for the AZO devices on Si/SiO2 wafer substrates. **c** The absorbance spectrum of the Zinc Oxide (ZnO) and AZO inks is limited in the Ultraviolet (UV) range to just over 350 nm, and does not sufficiently absorb over the visible wavelength. **d** The developed photodetectors (non-gated architecture) are tested in a potential divider configuration shown on the left, where a 10 V bias is applied to obtain the voltage (red line) and current (blue line) graph shown on the right. **e** The measurements from **d** are used to obtain the turn-on and turn-off times of a sample device. The blue line is the output current through a device based on a transient measurement. **f** Picture of the Ag interdigitated electrodes sputtered on a Si/SiO_2_ wafer (18 mm × 50 μm channel and 1 mm × 1 mm pad). **g** Picture of the device after inkjet printing the AZO ink. **h** Picture of a fully inkjet printed device. **i** A gated device (on a Si/SiO_2_ wafer) is tested under a 5 V bias for different gate voltages, and under different light conditions. **j** The current and on/off ratio of a simple device on glass with a thick layer (approx. 200 nm) of semiconductor.

For concept demonstration and with the above requirements in mind, we selected the following two architectures and materials for their ease of processing: (1) a simple planar metal-semiconductor-metal architecture on glass (Fig. 2a), and (2) a planar metal-semiconductor-metal architecture with a global gate on a Si/SiO2 wafer (Fig. 2b). The former offers a facile solution, easily manufacturable at scale, with only two main fabrication steps (electrodes deposition and semiconductor deposition). All the layers can be easily inkjet printed on demand for rapid prototyping and customisation of the photodetector design. The low-temperature process allows the glass to be replaced with a plastic substrate such as polyimide (PI) or polyethylene naphthalate (PEN). The second architecture is somewhat harder to translate to a flexible substrate consistently and reliably, since using a printable insulator, would add not only fabrication cost, but another point of failure with bending, stress, and insulator quality, complicating this simple and scalable solution.

The materials used in this experiment were Ag for the electrodes and Aluminum-doped ZnO (AZO) for their compatibility with inkjet printing and photosensitivity of the AZO (band diagram shown in Supplementary Fig. 1). Figure 2f, g shows the devices at various fabrication steps with sputtered Ag electrodes, while Fig. 2h is a fully printed photodetector.

### Oxide semiconductors – absorption

While the fabrication process is simple and well suited to the purpose, the optical properties of ZnO and AZO inks, as measured, are not aligned with our specific goals. As seen in Fig. 2c the semiconductors absorb mainly in the ultraviolet range, hence, ambient detection might be problematic and suboptimal. We performed measurements using a wide spectrum source to obtain basic electrical characteristics and assess the operation and suitability of the planar electrode structure. Moreover, there may be related applications, in which a dedicated light source might be utilised in combination with visible-blind photodetectors to achieve similar functionality. An example where this could be used is the third implementation of the hybrid Magic Bookmark which relies on the reflectance of light off the page and uses an array of dedicated light sources^23^. That approach has the best overall result in terms of the useability of the Magic Bookmark. However, no dedicated printed and flexible light-emitting device is explored in this work, thus the focus remains in implementing the second iteration of the system that relies on optical apertures. It is important to highlight the main electrical performance of the oxide devices, which could potentially open up opportunities for realising further user interface systems, purposefully taking advantage of the response which falls outside of the visible range.

### Oxide semiconductors – electrical characteristics of photodetectors

The measured electrical performance of the global-gate architecture shows a better controllable on/off ratio and an enhanced operation, compared to the simpler architecture on glass. The results in Fig. 2i, j, for example, show that a gated photodetector (Fig. 2i) at a 5 V drain to source bias (*V*_DS_) and a 20 V gate to source voltage (*V*_GS_) exhibit an on/off ratio 8 times higher than the on/off ratio of a simple metal-semiconductor-metal device (Fig. 2j) also at 5 V bias voltage and under the same light conditions (the gate refers to the Si global gate of the wafer, and the source and drain refer to the sputtered or printed electrodes that are interchangeable due to their symmetry). This is due to the effects of the gate, which induces a charge accumulation in the channel, thus improving the conductivity and enabling amplification. On the contrary, the simple non-gated device relies solely on the extraction of the photogenerated charges in the channel. Using the potential divider circuit shown in Fig. 2d-left to convert the current into voltage measurements, we obtained the outputs shown in Fig. 2d-right. The voltage difference of just under 200 mV for light vs dark conditions is more than sufficient for detecting the presence of light using a typical low power analogue to digital converter (for a detailed analysis see the “Organic Semiconductor Photodetectors” section below). The current output shown in Fig. 2d is also used to obtain the turn-off and turn-on times shown in Fig. 2e. At 1.6 s, turn-on time is acceptable for user interfaces, since these are usually low frequency applications that rely on human motions such as the manipulation, pressing or tapping of the sensor to trigger an action.

### Oxide semiconductors – suitability for the application

The simplicity of the symmetrical metal-semiconductor-metal structure both in terms of fabrication process and operation, makes it suitable for the application requirements outlined before. The devices can be easily printed on demand, which means they can be easily adapted in terms of size and shape for each application.

However, the absorbance mismatch of the material and the required spectrum is too large to ignore. The devices will be functional under very broad-spectrum illumination (i.e., solar radiation), but operation might be unnecessarily hindered for indoor light sources with a narrower emission spectrum over the visible range. Hence another more suitable semiconductor is explored next.

### Organic semiconductors – photodetectors structure

**Fig. 3:**
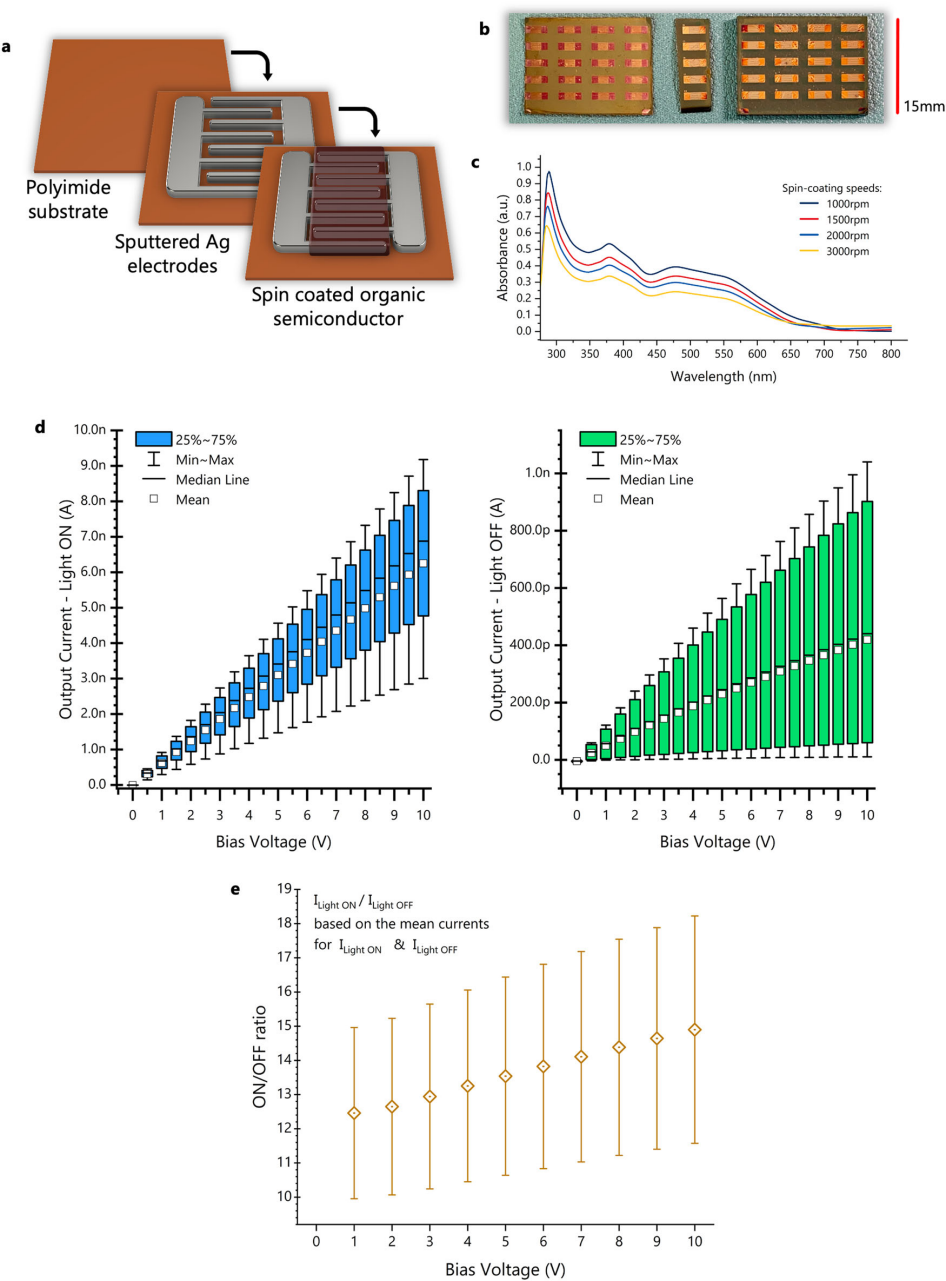
Electrical characterisation of the developed organic photodetectors on polyimide. **a** Fabrication process for the flexible photodetectors (the organic semiconductor blend is spin-coated on the substrate, graph shows semiconductor in the area of interest only). **b** Pictures of the array of devices (left: devices on polyimide, centre: devices on polyimide after cutting, right: control devices on glass substrates). **c** The absorbance spectrum of the organic semiconductor ink for different thicknesses (different spin speeds). **d** Statistical results from 20 photodetectors made on flexible polyimide substrate, measured with the light on (lamp setting 4E) and in the dark. **e** On/off ratio against bias voltage for flexible devices, based on the average on and off currents from 10 devices. The error bars show the maximum and minimum on/off ratios from the 10 devices.

**Fig. 4:**
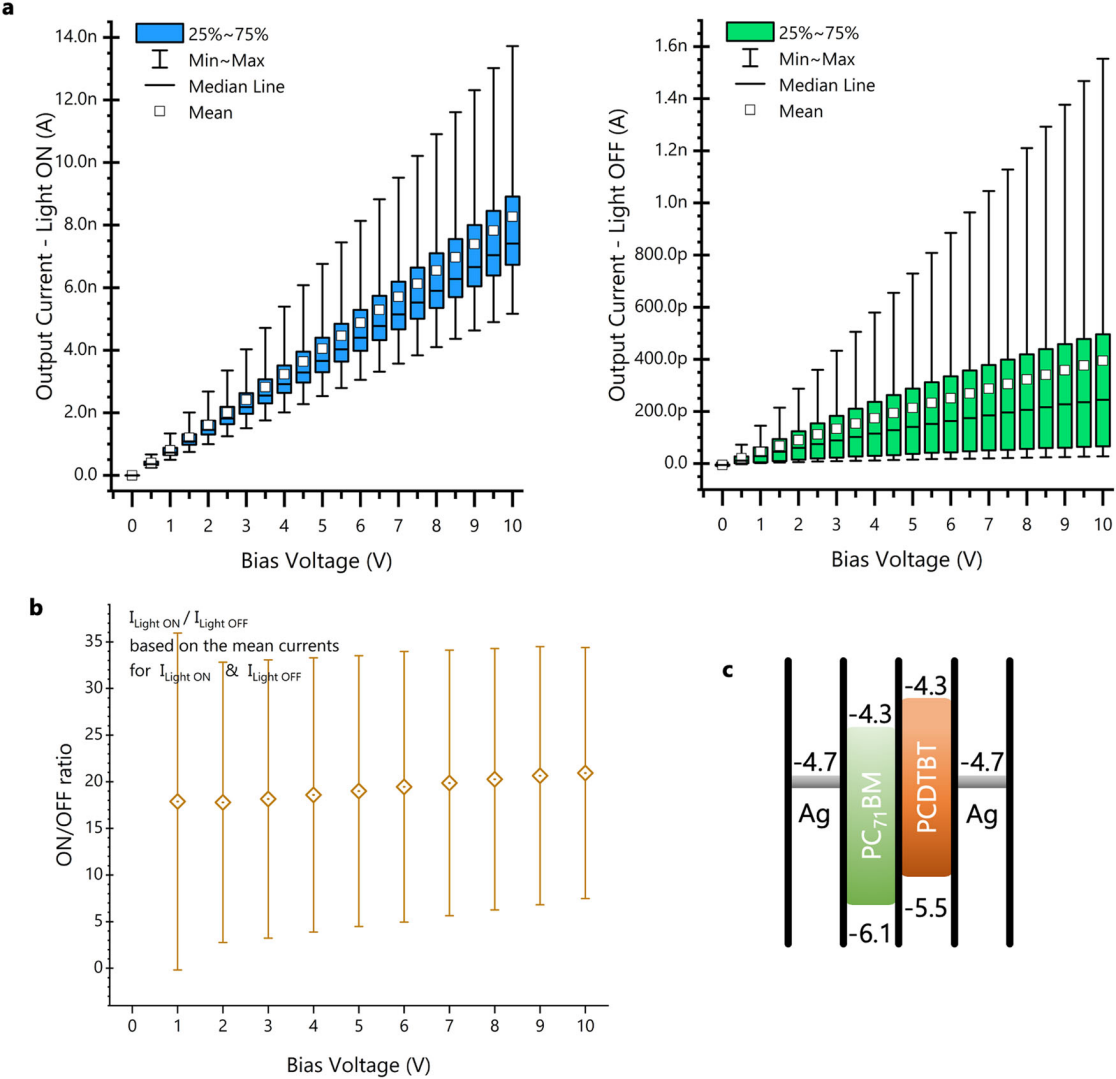
Electrical characterisation of the developed organic photodetectors on glass. **a** Statistical results from 20 photodetectors made on glass, measured with the light on (lamp setting 4E) and in the dark. **b** On/off ratio against bias voltage for devices on glass, based on the average on and off currents from 10 devices. The error bars show the maximum and minimum on/off ratios from the 10 devices. **c** Band diagram of the PCDTBT:PCBM metal-semiconductor-metal devices with Ag electrodes (all units in eV).

Based on the results obtained from the oxide semiconductor devices, the simple metal-semiconductor-metal structure has been selected for the next development phase. Organic semiconductors are more suitable for this application due to their intrinsic flexibility and their compatibility with solution processing. A PCDTBT:PCBM blend is deposited on top of the same interdigitated Ag electrode pattern used for the oxide semiconductor devices (band diagram shown in Fig. 4c). The devices are fabricated both on glass and flexible polyimide substrates to assess their performance after multiple bending cycles, using the glass devices as a benchmark. Figure 3a shows the two-step fabrication process for the organic photodetectors on flexible substrates, and Fig. 3b shows pictures of the fabricated arrays on polyimide (left and centre) and glass (right).

### Organic semiconductors – absorption

The absorption spectrum of the PCDTBT:PCBM blend is better suited to the application requirements, compared to ZnO and AZO. As shown in Fig. 3c the two materials complement each other absorbing over most of the visible wavelength, thus matching the spectrum of the test lamp (Fig. 1b). Additionally, the two materials chosen are a good donor/acceptor combination, which can be easy to process for correct phase separation ensuring sufficient charge separation and collection of the photogenerated carriers, despite the symmetrical electrode configuration (photoconductor configuration)^26^. Over time, it was observed that O_2_ further dopes the semiconductor, thus increasing the dark current and affecting the on/off ratio. This would explain any discrepancies in the on/off ratios mentioned between the experiments, as each was conducted at a different point in time on the same devices. Since the performance is sufficient for the application, as it will be discussed next, this observation is beyond the scope of this publication.

### Organic semiconductors – electrical characteristics of photodetectors

The electrical performance of the flexible photodetectors is investigated next. Statistical results were obtained from 20 devices, to assess the fabrication yield and the repeatability of the results. Figure 3d shows the variations of the on- and off-currents of the flexible detectors. The large spread in operating currents is largely linked to the non-uniform thickness of the semiconductor layer due to the vacuum-less spin coating of the flexible substrate. An indication of this limitation of flexible device processing is seen in Fig. 4a where the same statistics for devices on glass yield lower spread. For example, the 25–75% variation of the photogenerated current (light on) at 10 V bias voltage is 1.7 times smaller for the devices on glass compared to the ones on polyimide. On average, the on/off ratio is also better for the devices on glass with the ratio at 10 V bias (20.9 as shown in Fig. 4b) being 1.4 times higher compared to one for flexible devices (14.9 as shown in Fig. 3e). The turn-on and turn-off times of the detectors were also measured and provided in Supplementary Fig. 2.

Irrespective of the superior performance of the photodetectors on glass, the flexible devices can still perform sufficiently for the augmented paper ecosystem that they are being developed for. The average response obtained from the detectors was 1.05 nA/mW incident light intensity for a 5 V bias voltage with total active area of 1.54 mm^2^. This current is extremely small but is found to be sufficient for the desired functionality. In our scenario, the lighting should be adequate for a comfortable reading experience, so the system is unlikely to be required to operate in low incident light conditions. Nevertheless, even minimal optimisations to the cell architecture, material blend, or electrode work function should result in cells capable of successful page detection in very dim ambient lighting.

Let us consider an ATmega328 microcontroller with a 5 V supply, which is a common combination for many well-known development boards (e.g., Arduino UNO and Arduino Nano), together with the average current values obtained from the measured flexible photodetectors and a rudimentary current to voltage conversion scheme. With a dark current of *I*_Dark_ = 225 pA and a potential divider circuit (Fig. 6a) with a 10 MΩ resistor would result in a voltage drop of *V*_R_dark_ = 2.25 mV across the resistor. This drop is small compared to the 5 V supply and we can assume that the voltage across the device under test and thus the current through it will not be considerably affected. With a light-on current of *I*_Light_ = 3.1 nA the drop across the resistor would be *V*_R_light_ = 31 mV. The processor is equipped with 10-bit Analogue to Digital Converters (ADCs) and the simplest default configuration would be to use the 5 V rail as the reference voltage. This would result in steps of *V*_ADC_step_ = 5/2^10^ = 4.88 mV, which means that *V*_R_dark_ would be read as analogue ground (since 2.25/4.88 = 0.46 → 0 or step 0x00 i.e 0 V), and *V*_R_light_ would be read as 29.28 mV (in full: 31/4.88 = 6.35 → 6 or step 0x06, i.e., 4.88*6 = 29.28 mV). This means the measurement error is e = −5.5%, which can be further reduced to −3.3% if the internal 1.1 V ADC reference is used instead. It should be noted that the ADC is optimised for analogue signals with approximately 10 kΩ or less output impedance, hence the sensor output should be buffered before being measured to avoid loading the measurement circuit. This is easily achieved on the control side of the system.

**Fig. 5:**
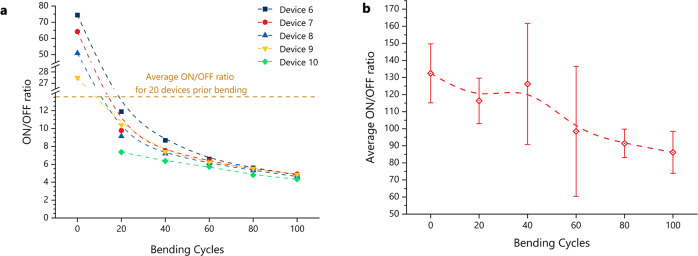
Bending performance of the flexible organic photodetectors. **a** On/off ratio of 5 flexible devices (with a 100 nm semiconductor) against 2 mm-diameter bending cycles, under a 5 V bias. The horizontal line indicates the average on/off ratio from 20 devices under a 5 V bias. **b** Average on/off ratio from 5 devices (with a thin 30 nm semiconductor layer) against 2 mm-diameter bending cycles. The error bars show the maximum and minimum on/off ratios from the 5 devices.

Important features of the developed photodetectors including their flexibility, reliability, and robustness are also assessed. The detectors have been repeatedly bent around a rod with a 1 mm radius 100 times, applying tensile stress, with the on/off currents being measured every 20 bending cycles. Two iterations of the devices have been tested, one with a thicker (~100 nm) semiconductor layer (results shown in Fig. 5a), and one with a thin (~30 nm) semiconductor layer (Fig. 5b). The 30 nm devices on/off ratio is superior because of the lower dark current, which is probably due to the thinner semiconductor layer, and the bending only starts affecting the results after 40 bending cycles. However, the on-currents are one order lower than the results obtained in Figs. 3d and 4a, hence the 100 nm structure was preferred.

Among the 100 nm devices, even the ones that show greater degradation with repeated bending can operate sufficiently with the microcontroller scenario presented above. Assuming the same dark current of 225 pA, with an on/off ratio of 5 (after 100 1 mm radius bending cycles), the current under illumination would be 1.13 nA (11.3 mV across the potential divider resistor), yielding step 0 × 0A (1.1 V reference), i.e., 10.74 mV, hence the measurement error would be −4.65%.

**Fig. 6:**
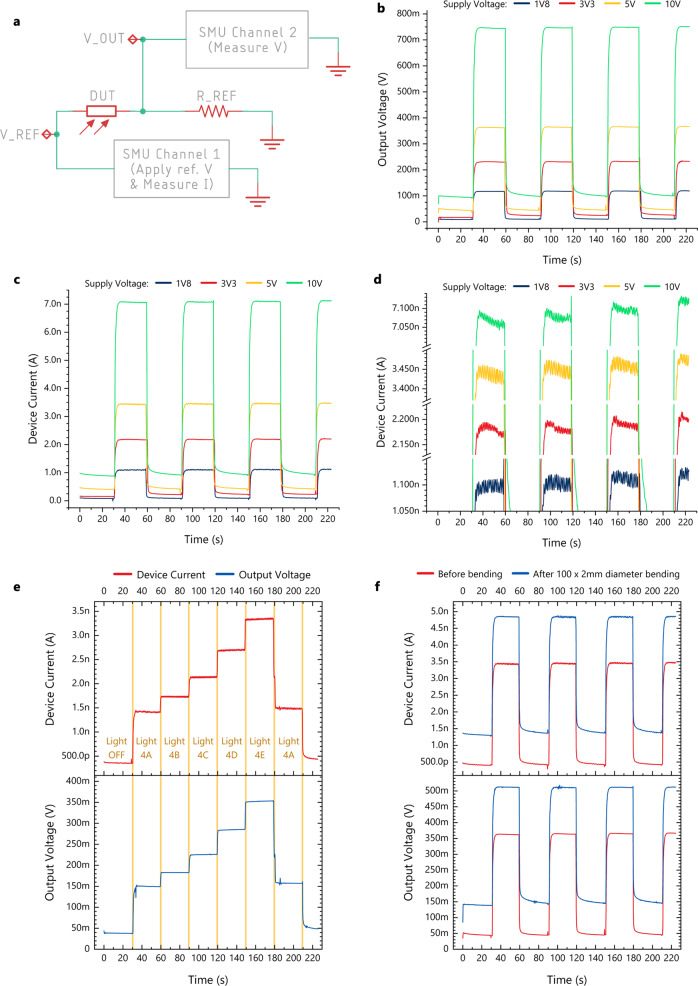
Real life electrical performance of the organic photodetectors. **a** Biasing circuit for the developed photodetectors, which can be used for incorporating the devices in an application. **b** The output voltage (Source Measurement Unit (SMU) channel 2) and **c** the output current (SMU channel 1) of circuit **a** under various supply voltages V_REF. **d** The output current shown in (**c**) is replotted on a different resolution to compare noise levels. As the supply voltage is increasing the noise is reduced. **e** Photodetector current and voltage from circuit (**a**) are measured while lighting conditions change (5 V supply voltage). **f** Photodetector current and voltage from circuit (**a**) are plotted for a single device, before and after 100 bending cycles with a 2 mm diameter (5 V supply voltage).

The above mathematical analysis is based on the average values for on and off currents, but lab tests were performed to validate it. The devices performed better than the calculations predict. Figure 6b, c shows the measured voltage and current respectively for different supply voltages. Seemingly, a higher supply voltage results in a better on current and hence, better expected performance. However, the ratio increases as the supply is reduced. In fact, the 10 V supply has a voltage on/off ratio of 7.76, the 5 V supply voltage 7.72, the 3.3 V supply 9.62, and the 1.8 V supply 11.80. The small output voltages, nonetheless, make the digitisation of the signal harder, and the noise larger (Fig. 6d). Therefore, a 5 V supply was chosen as a compromise which results in measurable and distinguishable quantities and is also a common microcontroller supply voltage. In fact, based on Fig. 6e, the 5 V supply allows even for clearly distinguishing between the different light settings of the test lamp (lamp settings in Supplementary Table 1). Even after multiple bending cycles, the real devices maintain useable performance, as shown in Fig. 6f; the voltage on/off ratio of 7.89 prior bending is reduced to 3.68, but remains distinguishable based on the theoretical calculations for the performance of the typical ADC discussed above.

### Organic semiconductors – suitability for the application

Similar to the oxide-based detectors, the simplicity of the metal-semiconductor-metal structure makes it easy to fabricate both on demand and on a large scale. The process is easy to implement on a flexible substrate, which is essential to the application, and the flexible device operation is robust and reliable even after multiple bending cycles. Additionally, the organic semiconductor blend shows excellent absorbance over the visible range required by augmented paper applications and user interfaces, and when used in a potential divider circuit to detect the presence of light the electrical output is sufficient. A fully printed implementation of the photodetectors and assessment of their stability is planned as the next step of the work, as the materials used are natively compatible with printing techniques.

Overall, the functionality offered by the developed flexible photodetectors is highly suitable for input sensors in user interface systems, such as the Magic Bookmark.

### Circuit integration

**Fig. 7:**
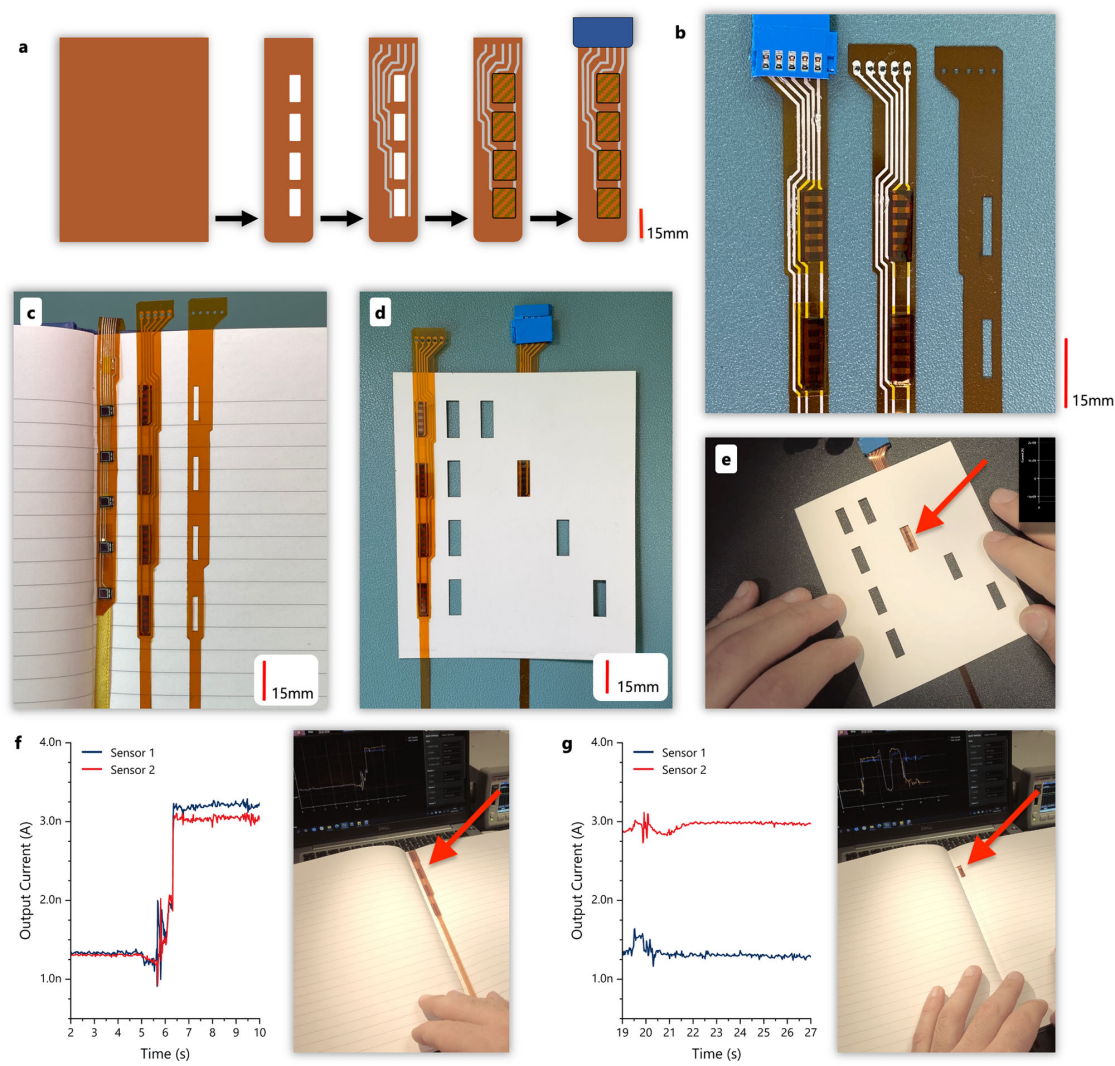
Integration process and pictures of the final prototype. **a** Outline of the photodetector integration process for the Magic Bookmark. **b** Pictures of the assembled Magic Bookmark in different stages of the process. **c** Different stages of the assembled Magic Bookmark next to its hybrid equivalent (using conventional photodiodes (left)). **d** A card with optical cutouts is used to test the final assembly. **e** Picture of the Magic Bookmark testing with the test card shown in (**d**). **f** Two sensors from this 4-sensor assembly are connected to the SMU. In this instance both sensors are exposed. **g** Following from (**f**) a page with an optical aperture that exposes only one of the sensors is placed on top of the bookmark. The blue and yellow lines on the top-tight of the picture shows that one sensor is exposed, and one is covered.

The integration of the developed devices with a functional system is necessary for evaluating the photodetectors under real-life conditions. Therefore, we used a scalable integration technique developed in-house for realising the Magic Bookmark system using the flexible organic photodetectors designed as part of this work. As shown in the diagram in Fig. 7a, a single sheet of polyimide is laser-cut to size and with the necessary optical apertures. An Ag-based paste is then dispensed on the cut substrate to realise the connections between the sensors and the electronic system. Before the paste is sintered, and while still in a liquid form, the sensors (previously cut to strips of 5) are flipped and positioned on top of the apertures. The Ag paste acts both as an electrical connection for the signal and as an adhesive. In the prototype, additional tape is used to hold the sensors in place and increase the system’s robustness. Finally, a standard connector is added at the end of the flexible bookmark strip to simplify the integration and the testing. Images of the various stages of the assembly process are shown in Fig. 7b. Figure 7c shows the evolution of the system, from a hybrid integration bookmark using conventional photodiodes (left) to the fully printable and flexible bookmark with the proprietary photodetectors (middle).

The current-to-voltage conversion requires a comparatively large value of R_REF (reference resistor of the potential divider, as shown in Fig. 6a). In principle, this could be realised on the flexible substrate itself by printing or dispensing high resistivity inks or pastes^27,28^. This approach would add another source of variability at a critical point within the electrical system and would require the V_REF signal to be routed to the flexible bookmark itself. In fact, carrying current rather than voltage information over the signal lines is preferred, and the inequal track length is inconsequential given the extremely low currents and adequate resistance (3.29 mΩ/sq) of the tracks.

The test card shown in Fig. 7d was used to expose and block the ambient light from the sensors manually, to test their operation under real-life conditions. Figure 7e shows the flexible bookmark being wrapped around a rod to demonstrate its flexibility. The final bookmark was also tested with a sample book that has optical apertures to expose the sensors (also see Supplementary Videos 1 and 2). Figure 7f shows the bookmark on top of the pages with all the sensors exposed. In this condition, both sensors output a high value when exposed to ambient light. Conversely, when only one sensor is exposed (Fig. 7g) the reading from sensor two is high, while that of sensor one is low. Therefore, a small number of sensors representing a binary code can be used to express the active page number, as required for the augmented paper ecosystem that is realised with this flexible Magic Bookmark prototype. Since the detectors can also detect multiple light levels, the encoding can be optimised is the application can partially obstruct the sensor, creating multilevel logic codes.

In terms of scalability for books with more pages, this implementation allows for at least 8 modules to be embedded on a typical A5 size book. Using photodetector groups of 5 results in 15 mm × 5 mm sensor modules per bit. Thus an 8-bit system capable of addressing 255 pages would require at least 120 mm plus additional spacing between modules, which can comfortably be accommodated within the 210 mm height of an A5 page. Removing the fabrication constrained imposed by the utilised shadow mask design would allow even higher integration rate.

## Conclusion

With augmented paper and the Magic Bookmark as a reference application, a set of key requirements for ambient light sensors was set, including reliability with bending, fabrication scalability, and absorption over the visible spectrum. The initial experiments with oxide semiconductors showed the fabrication benefits of a simple metal-semiconductor-metal structure, however the absorbance spectrum of the ZnO and AZO inks used was predictably unsuitable. Therefore, we formulated a common organic semiconductor blend (PCDTBT:PCBM) that was spin coated on top of coplanar Ag interdigitated electrodes. The photodetectors obtained by this simple process demonstrated an on/off ratio of 7.89, which dropped to 3.68 after 100 1 mm radius bending cycles. Considering a typical microcontroller (ATmega328) and its embedded ADC this is sufficient to detect the presence of light, and simultaneously different light levels, with an error as little as 3.3%. These results indicate a robust solution suitable for ambient light sensor arrays such as the ones needed for the Magic Bookmark.

The developed devices were integrated and tested in a prototype to demonstrate their suitability for scalable fabrication, which showed that variability of performance and electrical degradation with age and bending do not affect the functionality, as the system design of the Magic Bookmark allows for self-calibration, irrespective of the detector type, using a reference sensor. Therefore, we managed to design, fabricate and characterise fully-printable and flexible photodetectors specifically optimised for the requirements of real-life user interfaces.

Our approach shows that even extremely simple fabrication techniques and comparatively low-performing material systems can be viably utilised in large area flexible electronic applications, with the prerequisite of a system definition that critically considers both the advantages and the limitations of the functional devices.

## Methods

### Substrate preparation

Pre-cut 20 mm × 15 mm glass substrates are obtained from Ossila (product code S151). Polyimide substrates (75 um thickness) are obtained from RS Electronics (product code 536-3968). All substrates are cleaned in Acetone and IPA in an ultrasonic bath for 5’ in each solvent, followed by 5’ of oxygen plasma at 200 W. For the integration process, the polyimide sheets are cut using a Trotec Speedy300 Laser Engraver and Cutter at 50% power and 50% speed, and cleaned with IPA wipes.

### Ag electrodes

For inkjet printing, a Dimatix DMP2800 materials inkjet printer is used to deposit a JET600 (Hirose Electronics) Ag ink, followed by sintering at 120 °C for 30’. For the sputtered electrodes, a shadow mask from Ossila is used (product code E323), and Ag is sputtered for 20’ at 200 W to obtain a thick layer of Ag (~300 nm).

Oxide Semiconductor Deposition: The ZnO and AZO inks were manufactured by GenesInk (product codes H-SZ01034 and H-DZ01015 respectively) purchased from Sigma Aldrich. The Dimatix DMP2800 materials inkjet printer was used to deposit 3 layers of semiconductor for each device, followed by a slow ramp annealing (60 °C for 10’, 80 °C for 10’, 100 °C for 10’, 120 °C for 2 h).

### Organic semiconductor formulation

The organic semiconductor was formulated using the following materials: PCDTBT by Solaris (SOL4280L), [C70]PCBM by NanoC (PCBM71X09), 1,2-Dichlorobenzene by Sigma Aldrich (product code 240664), Chlorobenzene by Sigma Aldrich (product code 284513). The concentration of the solution is 14 mg of PCDTBT per ml of solvent mix and 56 mg of PCBM per ml of solvent mix, in solvent mix of 3:1 per volume of DCB:CB. The formulation is conducted in a nitrogen environment, followed by stirring for 7 days at room temperature.

### Organic semiconductor deposition

After the formulation is completed, the organic semiconductor blend is spin coated in ambient environment at 2000 rpm for 30”, which produces an approximately 110 nm thick layer of semiconductor. A 10 min annealing at 80 °C on a hot plate was done right after the spin coating.

### Electrical characterisation

The electrical characterisation is completed using a probe station and a Keysight B2902 2-channel source measurement unit. The light source is a Schott KL1500 LCD Cold Light Source, and its measured output power (using a Gentec P-Link (USB) power metre) for the various settings as seen from the photodetectors’ position is summarised in Supplementary Table 1. The options used in this work are highlighted in the table. The luminance intensity of the lamp for the various settings was also measured and is shown in Supplementary Table 2. Based on the reference values in the table, the testing conditions are less bright than full daylight conditions (not direct sun), but more intense compared to a reading lamp.

### Integration

A polyimide sheet is cut to size, creating light apertures. Ag paste is dispensed on substrate using a Voltera V-one printer. The photodetectors have been cut in groups of 5 that are connected in parallel. The devices are flipped on top of the uncured Ag paste, which is used for both a mechanical and an electrical connection. The assembly is then heated to 80 °C for 35’ (temperature limited by the organic semiconductor) to sinter the Ag paste. Finally, the devices can be encapsulated with another layer of tape for better mechanical stability and a connector is crimped on top of the assembly.

### Supplementary information

Sporea_PR File

Supplementary Information

Description of Additional Supplementary Files

Supplementary Movie 1

Supplementary Movie 2

## Data Availability

The datasets generated and analysed during the current study are available from the corresponding authors on reasonable request. Information about the resistance of the Voltera tracks can be found here: https://support.voltera.io/technical-data-sheets.
