# Supplementary material for: Printable and flexible photodetectors via scalable fabrication for reading applications
Source: Commun Eng. 2022 Dec 1;1:40. doi: 10.1038/s44172-022-00041-4 (PMC10956029; doi:10.1038/s44172-022-00041-4)
Supplement: Supplementary file 2 — Supplementary Information [file 44172_2022_41_MOESM2_ESM.pdf]

Printable and flexible photodetectors via scalable fabrication for reading applications

*Georgios Bairaktaris[1], Fasihullah Khan[1,2], K. D. J. Imalka Jayawardena[1], David M. Frohlich[3], Radu A. Sporea[1]**

1. Advanced Technology Institute, University of Surrey, Guildford, UK, GU2 7XH
2. Centre for Integrated semiconductors and Materials, Swansea University, UK, SA1
3. Digital World Research Centre, University of Surrey, Guildford, UK, GU2 7XH

* Corresponding Author's email address: r.a.sporea@surrey.ac.uk

Supplementary Figures

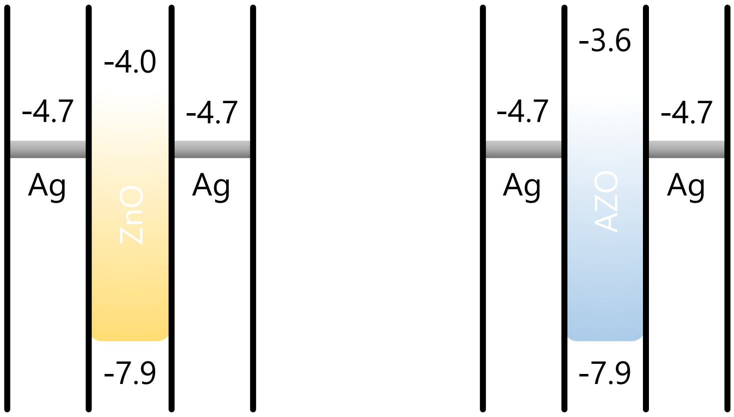

Supplementary Figure 1. Band diagram of the Zno (left) and AZO (right) metal-semiconductor-metal devices with Ag electrodes. All numbers correspond to eV units.

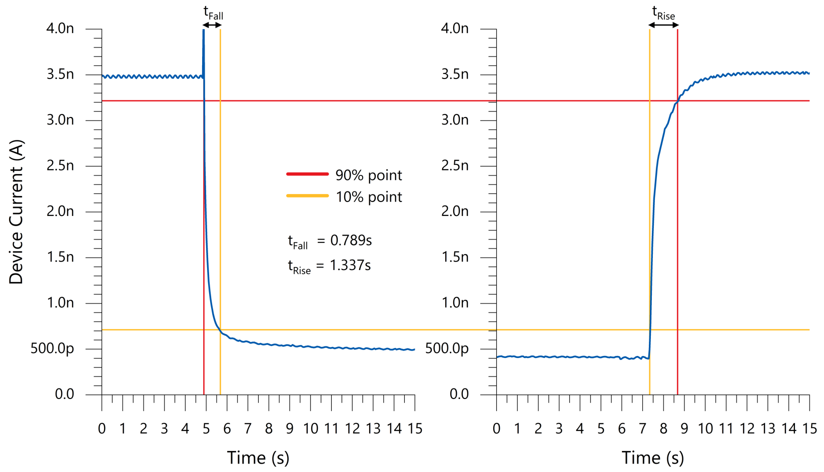

Supplementary Figure 2. Turn off and turn on times of the organic photodetectors developed on glass. The blue line on the figures is the device current obtained from a transient measurement.

Supplementary Tables

Supplementary Table 1. Measured output power (using a Gentec P-Link (USB) power meter) for the different settings of the Schott KL150 LCD lamp used to test the developed photodetectors. Settings 4A, 4B, 4C, 4D, and 4E are mainly used in this publication.

Columns: Lamp aperture (A-E) Rows: Light intensity setting (1-6)	A	B	C	D	E
1	110uW	171uW	299uW	571uW	1.01mW
2	103uW	189uW	408uW	753uW	1.40mW
3	146uW	284uW	574uW	1.18mW	2.16mW
4	222uW	374uW	773uW	1.59mW	2.95mW
5	328uW	686uW	1.27mW	2.57mW	4.85mW
6	388uW	842uW	1.62mW	3.16mW	6.02mW

Supplementary Table 2. Measured light intensity for the different settings of the Schott KL150 LCD lamp, measured using a Digital LUX meter LX1330B. Settings 4A, 4B, 4C, 4D, and 4E are mainly used in this publication. All the units are in lux. Some reference values are included at the bottom of the table.

Columns: Lamp aperture (A-E) Rows: Light intensity setting (1-6)	A	B	C	D	E
1	102	245	298	1054	2050
2	184	415	842	1680	3190
3	305	683	1340	2530	5280
4	457	967	2000	3900	7530
5	816	1617	3590	6410	13040
6	942	2180	4510	8970	16750
Laboratory light – measured at 30 cm = 4500 lux					
Reading lamp – measured at 30 cm = 1760 lux					
Sun overhead = 130000 lux [*1]					
Full daylight (indirect sun) = 10000 – 25000 lux [*1]					
Overcast day = 1000 lux [*1]					

Supplementary Videos

Supplementary Video 1. The final demonstrator is assembled with a book, that includes the necessary optical cut-outs to test the fully flexible and printed detectors within the Magic Bookmark ecosystem. Only the two middle detectors are connected to the SMU (due to the 2 available channels).

Supplementary Video 2. The final demonstrator that includes the developed photodetectors is tested with a test card. Only the two middle detectors are connected to the SMU (due to the 2 available channels).

Supplementary References

*1 Schlyter, Paul (1997–2009) [http://stjarnhimlen.se/comp/radfaq.html#10]
