# Supplementary material for: Printable and flexible photodetectors via scalable fabrication for reading applications
Source: Commun Eng. 2022 Dec 1;1:40. doi: 10.1038/s44172-022-00041-4 (PMC10956029; doi:10.1038/s44172-022-00041-4)
Supplement: Supplementary file 3 — Description of Additional Supplementary Files [file 44172_2022_41_MOESM3_ESM.pdf]

Description of Additional Supplementary Files

File name: Supplementary Movie 1

Description: The final demonstrator is assembled with a book, that includes the necessary optical cut-outs to test the fully flexible and printed detectors within the Magic Bookmark ecosystem. Only the two middle detectors are connected to the SMU (due to the 2 available channels).

File name: Supplementary Movie 2

Description: The final demonstrator that includes the developed photodetectors is tested with a test card. Only the two middle detectors are connected to the SMU (due to the 2 available channels).
