# Supplementary material for: Printable and flexible photodetectors via scalable fabrication for reading applications
Source: Commun Eng. 2022 Dec 1;1:40. doi: 10.1038/s44172-022-00041-4 (PMC10956029; doi:10.1038/s44172-022-00041-4)
Supplement: Supplementary file 1 — Sporea_PR File [file 44172_2022_41_MOESM1_ESM.pdf]

nature portfolio

Peer Review File

Minimally complex flexible organic photodetector arrays for scalable user interfaces and augmented reading

Open Access This file is licensed under a Creative Commons Attribution 4.0 International License, which permits use, sharing, adaptation, distribution and reproduction in any medium or format, as long as you give appropriate credit to the original author(s) and the source, provide a link to the Creative Commons license, and indicate if changes were made. In the cases where the authors are anonymous, such as is the case for the reports of anonymous peer reviewers, author attribution should be to 'Anonymous Referee' followed by a clear attribution to the source work. The images or other third party material in this file are included in the article's Creative Commons license, unless indicated otherwise in a credit line to the material. If material is not included in the article's Creative Commons license and your intended use is not permitted by statutory regulation or exceeds the permitted use, you will need to obtain permission directly from the copyright holder. To view a copy of this license, visit http://creativecommons.org/licenses/by/4.0/.

Reviewers' comments:

Reviewer #1 (Remarks to the Author):

The system proposed by the authors is a detachable "magic bookmark" which reads the unique markers on each page to give the book the opportunity to relay to a nearby smart device the page number of the active page, without special user intervention outside the gestures normally used during reading. The authors deliberately chose an extremely simple device and system architecture, including low-performance materials, to show the robustness of the detection approach, including the fact that high performance or high uniformity of the photdetector/photovoltaic cells is in fact not required. This should make a system that is extremely simple to make, and with further improvement, quite robust against mechanical bending stress, abrasion and material ageing. These results are important research for the social spread of printed electronics.

Comments:
The data and their explanations are satisfactory for publication.

I think that the manuscript will be even better if the following points are corrected or added.

1. It seemed that there was not enough description about the characteristics of a complete printing device using Ag ink. (Characteristics, etc.)

2. Many publications have more than 100 pages (7-8bit required). Is there a process limit for increasing the number of bits?

3. If sensors can be placed on both sides and light information from above and below can be obtained separately, I think it will be sufficient to detect half the number of pages. Is this possible? Please comment on the expandability as a sensor device.

Reviewer #2 (Remarks to the Author):

Bairaktaris, et al. demonstrate the design and fabrication of flexible photodetectors for a Magic Bookmark implementation. The high-level discussion regarding design constraints and performance requirements for this specific application is framed well. However, some of the specific results could be made more clear, and the demonstration stops short of a full implementation. The authors should revise the manuscript to address the following concerns prior to publication:

1. Some of the claims seem to be overstated and should be qualified based on the actual results. The title explicitly suggests a 'fully-printed' Magic Bookmark. The discussion of page 18 and the conclusion modifies this to 'fully printable,' which is technically more accurate, but for a manuscript focused on variability and manufacturing tolerances this leaves an important component lacking. My understanding is that the primary results included in the manuscript are for devices made with sputtered electrodes and a spin-coated organic photosensitive blend. Translating this to an actual fully-printed process is not trivial – that seems to be a primary point of this work. Without that final step, of realizing fully printed devices (and discussing the device design, etc. that can accommodate manufacturing variability to maintain functionality), the reported work seems in a sense incomplete. It would be interesting to have a fully-printed device and understand what factors (i.e., electrode gap, active layer thickness) contribute most to device variability and performance limitations.

2. The other incomplete-seeming aspect of the work is that all the characterization is performed with a high performance SMU. The authors do a good job explaining requirements and considerations for

implementing this with an Atmega328 (page 12-13), but don't (it seems) actually integrate the fabricated devices with such a microcontroller. Including this demonstration to validate that the results translate to a more practical implementation would strengthen the manuscript.

3. With the variety of samples and characterization, some of the reported results could be made more clear. For example, on page 14 and 21 the on/off is stated as 7.89, decreasing to 3.68 after 100 bending cycles. On page 4, the on/off is stated as 20.9. These values don't have any error/uncertainty included, which should be addressed (i.e., Figures 3f and 3i should have error bars). It appears that the 20.9 value lines up with figure 3i (i.e., for photodetectors on glass), so this doesn't apply to the flexible devices that the authors make (and therefore it's questionable to claim this value on page 4). The on/off reported as 7.89, dropping to 3.68 after bending cycles, appears to align with the data in Figure 4f, but not Figure 3j. Is there a reason for the discrepancy? Are there error bars from multiple measurements for this? Particularly considering the overarching theme, of design to accommodate manufacturing variability, a more thorough discussion of performance variation would be merited.

4. The main results appear to be collected with the light source at its highest setting. How does this compare to ambient light that would be representative of actual use? Only the relative intensity of different lamp settings is shown, there's no absolute intensity to generalize these results to arbitrary light levels. Supplementary Table 1 has output power, but not intensity.

5. The authors discuss stability to aging for the prototype, suggesting that electrical degradation with age (and bending) does not affect functionality. However, it's not clear that this data is included anywhere in the manuscript, or any quantitative analysis of this is discussed. Even if the on/off ratio is stable, a deterioration in both the on and off currents would affect how this interfaces with, e.g., a microcontroller (i.e., the very low current means only the low end of the ADC range is useful, so degradation in the on current could lead to failure to distinguish on and off states). As the authors mentioned, the small output voltages make digitization of the signal more difficult and noise more problematic.

Overall, this is an interesting manuscript that includes valuable discussion well-suited to diverse readers. I particularly applaud the authors' ability to make this accessible to readers from different fields relevant to printed electronics – i.e., materials, manufacturing, and device design – as those conversations across disciplines are much needed to move the field forward. If the authors can address the comments above with minor revisions, this could be made suitable for publication.

Reviewer #3 (Remarks to the Author):

Reviewing report for Communications Engineering Manuscript#: COMMSENG-22-0139-T

Criteria for publication

• The authors demonstrated usage of a cost-effective fabrication method, i.e., printing to realize the photodetectors for Magic Bookmark application. It's a part of authors continuous efforts (Refs.14-16) on realizing cost-effective and flexible augment application. This time the photodetectors were printed.
• The paper provides strong evidence for its conclusions
• The data are technically sound
• The manuscript is important to scientists in the field of Materials and Hybrid Integration. In a nutshell, the authors showed that with two printed photodetectors (both illuminated or one in shadow) can create a simple binary code (light/no-light corresponds to yes/no logical gate) to be used to express e.g., an active page number, required for the Magic Bookmark. Since the detectors can also detect multiple light levels, the encoding can be optimized, creating multilevel logic codes. This approach can be used for realizing various augment applications, which could be interesting for the communication engineering community.

The review

• The authors demonstrate cost-effective, fully-flexible printed ambient light photodetectors that can withstand over 100x bending cycles over a 1mm-radius (in Fig. 4f stands 2mm?) rod. The photodetectors were integrated in a two-step fabrication process, which can be up scalable. Realized 4-bit Magic Bookmark prototype is used as an interface detector for an ambient/indoor light. So far authors reported (Refs. 14 – 16) photodetectors based on a hybrid approach embedding conventional photodiodes on a foil. This time they realized printed photodetector array.

• Authors refer this work as an output of continuous efforts on realizing low-cost, flexible augmentation concept. Previous work, reported in Ref. 14-16, was updated here by deploying printing and simply assembly fabrication process to realize the photodetector. They used a planar photoconductor configuration, coated organic semiconductor from a solution to develop thin film, a photosensitive active layer. Printed photodetectors were coupled with analog-to-digital converter to read the output. i.e., light/no-light signal. Using printed photodetectors, the authors realized Magic Bookmark augment prototype. As shown in Fig. 5c (this work), authors showed chronology in progress distinguishing prior and current photodetector state of the art (this work). Figure 5f, shows the bookmark on top of the book's page with two sensors exposed. A sufficient output was observed when both sensors were exposed to low intensity ambient/indoor light. This simple photodetector configuration (interdigital electrodes coated with solution processed semiconductor) results in useful device for complex augment application.

On the other side, hybrid integration of the printed photodetectors on FCBs were previously reported in Sun Sensor study. (Marjanović, N. et al. (2018). Hybrid Integration; Case Study with Sun Sensor for Cube Satellites. In: Meboldt, M., Klahn, C. (eds) Industrializing Additive Manufacturing - Proceedings of Additive Manufacturing in Products and Applications - AMPA2017. AMPA 2017. Springer, Cham. https://doi.org/10.1007/978-3-319-66866-6_25), In that study, photodiodes were inkjet printed and staggered configuration and adjusted for Sun Sensor application.

• The paper can be of interest to others in the fields
• The paper can influence thinking in the field - engineers
• The claims are convincing. However, a part with oxide semiconductor (ZnO) can be shortened. This output can be mentioned, but not in details.
• No other experiments needed
• The claims are appropriately discussed in the context of previous literature
• The manuscript is acceptable in present form (with minor adjustment as suggested, title, additional citations)

Other questions for referees to consider

• The manuscript is clearly written. In a nutshell, the authors realized a fully-flexible Magic Bookmark, rather than a "fully-printed photodetector arrays". As a suggestion, small adjustment in the title would better reflect essence of the paper.
• The manuscript can be shortened to aid communication of the most important findings. The part describing in details Oxide based semiconductor photodetectors is not necessary to be in a full length, i.e., Fig.2. Authors can mention that they fabricate and characterized them, as a benchmarking device, but since absorption spectra weakly match with visible part of the ambient light spectra, further study was omitted.
• The authors done themselves justice without overselling their claims
• They can cite previous work done in hybrid integration previously reported. Also, many new dated citations. There are references addressing hybrid integration approach from earlier dates (e.g., 2017, 2018), as well as organic photodetectors (early 2005 up today), which would give the impression that the field is a bit older than presented.
• The authors provided sufficient methodological detail that the experiments could be reproduced
• The statistical analysis of the data is sound
• No further data or methodological information needed
• No special ethical concerns arises

University of Surrey
Guildford, Surrey, UK
30 August 2022

Realising the fully-printed Magic Bookmark:
minimally complex flexible photodetector arrays for scalable user interfaces

Georgios Bairaktaris, Fasihullah Khan, K. D. J. Imalka Jayawardena,
*David M. Frohlich, Radu A. Sporea**

Herein, we are submitting a revision of the manuscript under the revised title:

Realising the fully-printable Magic Bookmark:
minimally complex flexible photodetector arrays for scalable user interfaces

The reviewers' comments are addressed below:

Reviewer #1 (Remarks to the Author):

> The system proposed by the authors is a detachable "magic bookmark" which reads the unique markers on each page to give the book the opportunity to relay to a nearby smart device the page number of the active page, without special user intervention outside the gestures normally used during reading. The authors deliberately chose an extremely simple device and system architecture, including low-performance materials, to show the robustness of the detection approach, including the fact that high performance or high uniformity of the photodetector/photovoltaic cells is in fact not required. This should make a system that is extremely simple to make, and with further improvement, quite robust against mechanical bending stress, abrasion and material ageing. These results are important research for the social spread of printed electronics.

We would like to sincerely thank the reviewer for their feedback and for highlighting the importance of the work for the social spread of emerging technologies such as printed electronics.

> Comments:
> The data and their explanations are satisfactory for publication.
>
> I think that the manuscript will be even better if the following points are corrected or added.
>
> 1. It seemed that there was not enough description about the characteristics of a complete printing device using Ag ink. (Characteristics, etc.)

Due to equipment limitations, we chose to realise the present demonstration comprising organic photodetectors with techniques which are not fully printed. However, all the materials (Ag precursors or suspensions for electrodes, and organic semiconductor solution) are compatible with printing techniques. We prepared the current paper as a report of our approach centred on the observation that even low-efficiency photodetectors with simple fabrication can be sufficient for the success of the application. This work is currently in progress, aiming for a fully-printed detection array and interconnect, a significant undertaking in itself which will be treated comprehensively in a subsequent submission. We have also added a relevant statement on page "xv" of the manuscript.

> 2. Many publications have more than 100 pages (7-8bit required). Is there a process limit for increasing the number of bits?

The integrated design is using photodetector groups of 5, resulting in a small 15mm x 5mm array per bit. Thus an 8-bit system would require at least 8 x 15 = 120mm plus additional spacing in between each sensor module. A5 paper height is 210mm which allows for (210 – 120)/8 = 11.25mm maximum spacing between

each sensor module that is more than sufficient for distinguishing the sensors and creating the apertures. The detection method also uses only $n+1$ interconnect paths for 2^n-1 distinct codes (to account for the ground lead and a standby state which does not represent any page in the book). In previous work, we have also shown that the different interconnect resistance does not play a role in the detection accuracy given the low current levels. Consequently, a 100-page book could be fully instrumented without difficulty with an array of 7/8 photodetectors and 8/9 interconnect traces. This analysis has also been appended on the manuscript (page "xix").

> 3. If sensors can be placed on both sides and light information from above and below can be obtained separately, I think it will be sufficient to detect half the number of pages. Is this possible? Please comment on the expandability as a sensor device.

This is a very interesting thought, thank you. A comment on the expandability of the system to detect a larger number of pages has been added. Since this Magic Bookmark technique required the system to be placed after the active page, the bottom side is covered by itself so light detection would not be beneficial. We do appreciate the reviewer's comment, however, as this would allow for further user inputs. In particular, if the sensors are combined with a light-emitting device, the third implementation of Magic Bookmark (https://doi.org/10.1002/aisy.202100138), in which an optical pattern similar to a barcode is read directly on the active page, would be feasible, and therefore integrating these simple photodetectors on the top side the bookmark would allow for user inputs, similar to touch sensitive buttons to be used. In our idea generation sessions, we have explored numerous additional functionalities which could be added to the bookmark. Here, in the interest of demonstrating a self-contained, viable system, we focused exclusively on the page detection, and in one of the three implementations proposed previously.

Once again, we would like to thank the reviewer for analysing and commenting on our manuscript.

> Reviewer #2 (Remarks to the Author):
>
> Bairaktaris, et al. demonstrate the design and fabrication of flexible photodetectors for a Magic Bookmark implementation. The high-level discussion regarding design constraints and performance requirements for this specific application is framed well. However, some of the specific results could be made more clear, and the demonstration stops short of a full implementation. The authors should revise the manuscript to address the following concerns prior to publication:

We would like to thank the reviewer for their time and comments, that we have individually addressed below.

> 1. Some of the claims seem to be overstated and should be qualified based on the actual results. The title explicitly suggests a 'fully-printed' Magic Bookmark. The discussion of page 18 and the conclusion modifies this to 'fully printable,' which is technically more accurate, but for a manuscript focused on variability and manufacturing tolerances this leaves an important component lacking. My understanding is that the primary results included in the manuscript are for devices made with sputtered electrodes and a spin-coated organic photosensitive blend. Translating this to an actual fully-printed process is not trivial – that seems to be a primary point of this work. Without that final step, of realizing fully printed devices (and discussing the device design, etc. that can accommodate manufacturing variability to maintain functionality), the reported work seems in a sense incomplete. It would be interesting to have a fully-printed device and understand what factors (i.e., electrode gap, active layer thickness) contribute most to device variability and performance limitations.

We understand the reviewer's concern and agree objectively to their view. This report is meant as an account of progress toward a system that removes the off the shelf chips from the design, for a much more practical implementation, considering the application. We demonstrate that the semiconductor solution processing route is viable, with extremely simple "planar" photosensors demonstrating the required properties. The material choice for electrodes (Ag) gives us confidence that transferring to an inkjet printing process has a good chance of success despite obvious departures in surface morphology and somewhat different interface

properties. In fact, our current work is advancing in that direction very successfully, and we will be publishing with a focus on the aspects of printing as a completely separate study, including systematic variations in ink formulation and printing process parameters.

We have added a statement in the manuscript (page "xv") and amended the title as suggested.

> 2. The other incomplete-seeming aspect of the work is that all the characterization is performed with a high performance SMU. The authors do a good job explaining requirements and considerations for implementing this with an Atmega328 (page 12-13), but don't (it seems) actually integrate the fabricated devices with such a microcontroller. Including this demonstration to validate that the results translate to a more practical implementation would strengthen the manuscript.

We understand the reviewer's suggestion. The scope of this work is to assess the suitability of printed electronic techniques and low-level emerging devices for the practical implementation of real-life systems. Therefore, we did not pursue the higher-level implementation of the system with off-the-shelf development boards. We have performed this integration many times for both analog and digital read-out of photosensor output. As such, we considered that replicating that part of the work would be of minimal benefit and focused on the printed/flexible electronics aspect of the system instead. Examples where we have demonstrated a fully integrated system are below:

1. "Next generation paper: an augmented book platform", Proceedings Volume 10738, Organic and Hybrid Sensors and Bioelectronics XI; 1073811, September 2018, https://doi.org/10.1117/12.2320107
2. "Printed Light Tags and the Magic Bookmark: Using light to augment paper objects", Extended Abstracts of the 2021 CHI Conference on Human Factors in Computing Systems, May 2021, https://doi.org/10.1145/3411763.3451566
3. "Magic Bookmark: A Nonintrusive Electronic System for Functionalizing Physical Books", Advanced Intelligent Systems, October 2021, https://doi.org/10.1002/aisy.202100138

> 3. With the variety of samples and characterization, some of the reported results could be made more clear. For example, on page 14 and 21 the on/off is stated as 7.89, decreasing to 3.68 after 100 bending cycles. On page 4, the on/off is stated as 20.9. These values don't have any error/uncertainty included, which should be addressed (i.e., Figures 3f and 3i should have error bars). It appears that the 20.9 value lines up with figure 3i (i.e., for photodetectors on glass), so this doesn't apply to the flexible devices that the authors make (and therefore it's questionable to claim this value on page 4). The on/off reported as 7.89, dropping to 3.68 after bending cycles, appears to align with the data in Figure 4f, but not Figure 3j. Is there a reason for the discrepancy? Are there error bars from multiple measurements for this? Particularly considering the overarching theme, of design to accommodate manufacturing variability, a more thorough discussion of performance variation would be merited.

We thank the reviewer for the suggestions and error bars have been added on Figures 3f and 3i as recommended. As explained above, since submission, we have been expanding this work further, realising fully printed photodetectors and studying their stability in air. Our latest findings show that O_2 further dopes the semiconductor over time, thus increasing the dark current and reducing the on/off ratio. This explains the discrepancy in the on/off ratio throughout the manuscript as each experiment was conducted at a different point in time on the same devices. While this statement may be concerning given the stated use case, the on/off ratio of the signal is still maintained to values which allow reliable detection (relevant information included on page "xi" of the manuscript). These results will be included in the next publication for which the work is currently in progress.

> 4. The main results appear to be collected with the light source at its highest setting. How does this compare to ambient light that would be representative of actual use? Only the relative intensity of different lamp settings is shown, there's no absolute intensity to generalize these results to arbitrary light levels. Supplementary Table 1 has output power, but not intensity.

We thank the reviewer for this important observation. In considering the application to augmented books, we debated several approaches to detecting the incident light. Here, we are using the resulting current while the

structure is biased, but other options for detection include simply sensing the open-circuit voltage of a photovoltaic cells. Recent advances in indoor photovoltaics show that V_{OC} can be reasonably high even at low ambient light levels. In that sense, the architecture of our system permits the use of the most suitable technology.

In this study, we took the simplicity of photosensor realisation to an extreme, and showed that operation is satisfactory using a light source which is comparable to direct indoor lighting. Our setup allowed us to measure the light emission profile and the output power, but nor the intensity directly. The test lamp is set in setting 4 out of 6, the aperture fully opened, and the measurements taken just under 15 cm below the end of the waveguide, at the same position where the devices would be placed during the experiment. A simple comparison shows that the intensity of typical lamps will be sufficient for triggering the photodetector's output.

5. The authors discuss stability to aging for the prototype, suggesting that electrical degradation with age (and bending) does not affect functionality. However, it's not clear that this data is included anywhere in the manuscript, or any quantitative analysis of this is discussed. Even if the on/off ratio is stable, a deterioration in both the on and off currents would affect how this interfaces with, e.g., a microcontroller (i.e., the very low current means only the low end of the ADC range is useful, so degradation in the on current could lead to failure to distinguish on and off states). As the authors mentioned, the small output voltages make digitization of the signal more difficult and noise more problematic.

We share the reviewer's interest in stability and have been extensively exploring this as part of the next step of the work presented in the manuscript. Our results indicate that both dark and light currents are increased after exposure to ambient environment with the on/off ratio dropping by just under 1 order of magnitude (from 80 to 10 after 3 weeks exposure to O_2 without any gas barrier encapsulation). This is still work in progress. Regarding the drop in performance, even the lowest on/off ratio allows for sufficiently distinguishing the light-on and light-off states, as per the calculations in the manuscript. The resistor values can be chosen so that the minimum expected voltage in low light condition and after degradation can be read reliably. There is no concern in the application if the top end of the scale saturates the input of the ADC for fresh photodetectors exposed to high intensity, and the threshold for detection can be calibrated after some experiments. Another solution is to use a self-calibrating design which uses an extra photodetector. Assuming reasonable performance variation between the sensors on each bookmark, this could be used as a reference light intensity monitor. We have not implemented this approach as we have demonstrated the concept functionally some time ago (https://vimeo.com/156681611).

Overall, this is an interesting manuscript that includes valuable discussion well-suited to diverse readers. I particularly applaud the authors' ability to make this accessible to readers from different fields relevant to printed electronics – i.e., materials, manufacturing, and device design – as those conversations across disciplines are much needed to move the field forward. If the authors can address the comments above with minor revisions, this could be made suitable for publication.

Once again, we would like to sincerely thank the reviewer for their time and hope that the above comments address the comments sufficiently. We agree with the observations and suggestions. In fact, the declared purpose of the technological development in our Next Generation Paper project is to bring together solutions that work cohesively for an application that has cost, reliability, form factor and ageing concerns. Hence our preoccupation for demonstrating, in stages, the practicality of various approaches (here, the low-complexity sensors with solution processing toward fully printed implementations). We have been working on these additional items extensively over the intervening period, in preparation for a subsequent focused publication that will show a fully-printed implementation and a stability assessment.

Reviewer #3 (Remarks to the Author):

Reviewing report for Communications Engineering Manuscript#: COMMSENG-22-0139-T

Criteria for publication

• The authors demonstrated usage of a cost-effective fabrication method, i.e., printing to realize the photodetectors for Magic Bookmark application. It's a part of authors continuous efforts (Refs.14-16) on realizing cost-effective and flexible augment application. This time the photodetectors were printed.
• The paper provides strong evidence for its conclusions
• The data are technically sound
• The manuscript is important to scientists in the field of Materials and Hybrid Integration. In a nutshell, the authors showed that with two printed photodetectors (both illuminated or one in shadow) can create a simple binary code (light/no-light corresponds to yes/no logical gate) to be used to express e.g., an active page number, required for the Magic Bookmark. Since the detectors can also detect multiple light levels, the encoding can be optimized, creating multilevel logic codes. This approach can be used for realizing various augment applications, which could be interesting for the communication engineering community.

We thank the reviewer for taking the time to thoroughly read and review our manuscript and have addressed their feedback below.

The review
• The authors demonstrate cost-effective, fully-flexible printed ambient light photodetectors that can withstand over 100x bending cycles over a 1mm-radius (in Fig. 4f stands 2mm?) rod. The photodetectors were integrated in a two-step fabrication process, which can be up scalable. Realized 4-bit Magic Bookmark prototype is used as an interface detector for an ambient/indoor light. So far authors reported (Refs. 14 – 16) photodetectors based on a hybrid approach embedding conventional photodiodes on a foil. This time they realized printed photodetector array.

• Authors refer this work as an output of continuous efforts on realizing low-cost, flexible augmentation concept. Previous work, reported in Ref. 14-16, was updated here by deploying printing and simply assembly fabrication process to realize the photodetector. They used a planar photoconductor configuration, coated organic semiconductor from a solution to develop thin film, a photosensitive active layer. Printed photodetectors were coupled with analog-to-digital converter to read the output. i.e., light/no-light signal. Using printed photodetectors, the authors realized Magic Bookmark augment prototype. As shown in Fig. 5c (this work), authors showed chronology in progress distinguishing prior and current photodetector state of the art (this work). Figure 5f, shows the bookmark on top of the book's page with two sensors exposed. A sufficient output was observed when both sensors were exposed to low intensity ambient/indoor light. This simple photodetector configuration (interdigital electrodes coated with solution processed semiconductor) results in useful device for complex augment application.

On the other side, hybrid integration of the printed photodetectors on FCBs were previously reported in Sun Sensor study. (Marjanović, N. et al. (2018). Hybrid Integration; Case Study with Sun Sensor for Cube Satellites. In: Meboldt, M., Klahn, C. (eds) Industrializing Additive Manufacturing - Proceedings of Additive Manufacturing in Products and Applications - AMPA2017. AMPA 2017. Springer, Cham. https://doi.org/10.1007/978-3-319-66866-6_25), In that study, photodiodes were inkjet printed and staggered configuration and adjusted for Sun Sensor application.
• The paper can be of interest to others in the fields
• The paper can influence thinking in the field - engineers
• The claims are convincing. However, a part with oxide semiconductor (ZnO) can be shortened. This output can be mentioned, but not in details.
• No other experiments needed
• The claims are appropriately discussed in the context of previous literature
• The manuscript is acceptable in present form (with minor adjustment as suggested, title, additional citations)

Other questions for referees to consider

• The manuscript is clearly written. In a nutshell, the authors realized a fully-flexible Magic Bookmark, rather than a "fully-printed photodetector arrays". As a suggestion, small adjustment in the title would better reflect essence of the paper.

We have changed the title based on the comments from Reviewer 2 and 3 to better reflect the implementation of the photodetectors in this work.

• The manuscript can be shortened to aid communication of the most important findings. The part describing in details Oxide based semiconductor photodetectors is not necessary to be in a full length, i.e., Fig.2. Authors can mention that they fabricate and characterized them, as a benchmarking device, but since absorption spectra weakly match with visible part of the ambient light spectra, further study was omitted.

We have chosen to include the oxide semiconductor section for its potential use in a different implementation of the Magic Bookmark. The third technique of the Magic Bookmark, as outlined in our publication "Bairaktaris, G. et al. Magic Bookmark: A Nonintrusive Electronic System for Functionalizing Physical Books. Advanced Intelligent Systems 4, 2100138 (2022)", https://doi.org/10.1002/aisy.202100138 , is using a combination of photodetectors and light-emitting devices to detect the reflectance of the page and thus a barcode style code. The oxide semiconductor could be potentially used for such a system if a printed light-emitting device could also be incorporated on the bookmark. To be viable for the declared intent of the system (i.e. a major requirement or the implementation to keep the distraction to the user to a minimum), visible light would not be practical in this approach. The system would need to be solar-blind to avoid false page detection and would generate light outside the visible range for the sake of the user's reading experience. Since we have not explored the emission part of such potential printed systems, we chose to focus mainly on the organic photodetectors, but believe it is worth discussing the oxide solutions for completeness. We have expanded this explanation further on the relevant part of the manuscript (page "viii").

• The authors done themselves justice without overselling their claims
• They can cite previous work done in hybrid integration previously reported. Also, many new dated citations. There are references addressing hybrid integration approach from earlier dates (e.g., 2017, 2018), as well as organic photodetectors (early 2005 up today), which would give the impression that the field is a bit older than presented.

We expanded the reference list to include more up-to-date publications in the fields of hybrid integration, user interfaces, and printed photodetectors.

• The authors provided sufficient methodological detail that the experiments could be reproduced
• The statistical analysis of the data is sound
• No further data or methodological information needed
• No special ethical concerns arises

We sincerely thank the reviewer for their feedback and encouraging comments.

Sincerely yours,

Dr Radu Sporea, SMIEEE MIET

Senior Lecturer in Semiconductor Devices
Advanced Technology Institute, University of Surrey
Guildford GU2 7XH, United Kingdom
r.a.sporea@surrey.ac.uk +44 1483 686086

on behalf of all authors.

Reviewers' comments:

Reviewer #1 (Remarks to the Author):

The submitted manuscript was appropriately revised and the content of the manuscript was enhanced. The author's manuscript is considered suitable for publication.

Reviewer #2 (Remarks to the Author):

The authors have addressed most of my original comments. I do maintain that some characterization of the light intensity would be valuable. Most researchers working on photodetector materials/devices report a sensitivity (for example, nA reading per mW light intensity, both normalized to area). Knowing what sensitivity is necessary for a device would be valuable for those researchers, and some discussion of matching that device sensitivity to a particular device area to get the output current within a suitable range would also be helpful. Even a decent estimate of the light intensity that corresponds to the different lighting levels used here would go a long way to generalize these findings and provide an important quantitative element to the work. Aside from that, it is an interesting manuscript and well-written.

Reviewer #3 (Remarks to the Author):

The authors adjusted the revised manuscript as suggested previously. In particular, they:
1. adjusted the title, better reflecting the presented work
2. clarified extended potential of the oxide semiconductors on page viii. By adding that clarification, the oxide part may stay in the text.
3. added extended list of references – now better covering state of the art in the hybrid electronics domain.

University of Surrey
Guildford, Surrey, UK
26 September 2022

Realising the fully-printable Magic Bookmark:
minimally complex flexible photodetector arrays for scalable user interfaces

Georgios Bairaktaris, Fasihullah Khan, K. D. J. Imalka Jayawardena,
*David M. Frohlich, Radu A. Sporea**

Herein, we are submitting a revision of the manuscript. The reviewers' comments are addressed below:

> Reviewer #1 (Remarks to the Author):
>
> The submitted manuscript was appropriately revised and the content of the manuscript was enhanced. The author's manuscript is considered suitable for publication.

We sincerely thank the reviewer for commenting on our manuscript and we appreciate their time.

> Reviewer #2 (Remarks to the Author):
>
> The authors have addressed most of my original comments. I do maintain that some characterization of the light intensity would be valuable. Most researchers working on photodetector materials/devices report a sensitivity (for example, nA reading per mW light intensity, both normalized to area). Knowing what sensitivity is necessary for a device would be valuable for those researchers, and some discussion of matching that device sensitivity to a particular device area to get the output current within a suitable range would also be helpful. Even a decent estimate of the light intensity that corresponds to the different lighting levels used here would go a long way to generalize these findings and provide an important quantitative element to the work. Aside from that, it is an interesting manuscript and well-written.

We would like to thank the reviewer for their time, and we share their concern regarding the light characterisation. As suggested, we measured the light intensity of the test lamp using a digital lux meter. The results are added as Supplementary Table 2, which also includes some reference values. This table is also referred to in the Methods section of the main manuscript. It appears that the main setting used, 4E, is brighter than a typical lamp, but remains less intense than full daylight conditions (indirect sun). However, it has been shown in Figure 4 that even less bright settings, such as 4A, can also yield recognisable output currents. Overall, the performance of the detectors is sufficient to function with indoor lighting, but could be further optimised for less bright conditions. Our goal was to show that this relatively crude method is suitable for robust detection under reasonable lighting conditions. Naturally, the active materials, detector area, and its geometry can be tailored to specific application needs.

We hope that these data are sufficient to address the reviewer's concerns, by indicating the light levels for which these photodetectors are functional. Once again, we thank the reviewer for the comprehensive review of our manuscript.

> Reviewer #3 (Remarks to the Author):
>
> The authors adjusted the revised manuscript as suggested previously. In particular, they:
> 1. adjusted the title, better reflecting the presented work
> 2. clarified extended potential of the oxide semiconductors on page viii. By adding that clarification, the oxide part may stay in the text.

3. added extended list of references – now better covering state of the art in the hybrid electronics domain.

We would like to thank the reviewer for analysing and commenting on the revised manuscript.

Sincerely yours,

Dr Radu Sporea, SMIEEE MIET

Senior Lecturer in Semiconductor Devices
Advanced Technology Institute, University of Surrey
Guildford GU2 7XH, United Kingdom
r.a.sporea@surrey.ac.uk +44 1483 686086

on behalf of all authors.

REVIEWERS' COMMENTS:

Reviewer #2 (Remarks to the Author):

The authors have addressed all of my concerns. It is an interesting paper, and well-written.
